# Supplementary material for: Characterization of a Novel L-Asparaginase from Mycobacterium gordonae with Acrylamide Mitigation Potential
Source: Foods. 2021 Nov 16;10(11):2819. doi: 10.3390/foods10112819 (PMC8617759; doi:10.3390/foods10112819)
Supplement: Supplementary file 1 [file foods-10-02819-s001.zip › foods-1451040-supplementary.pdf]

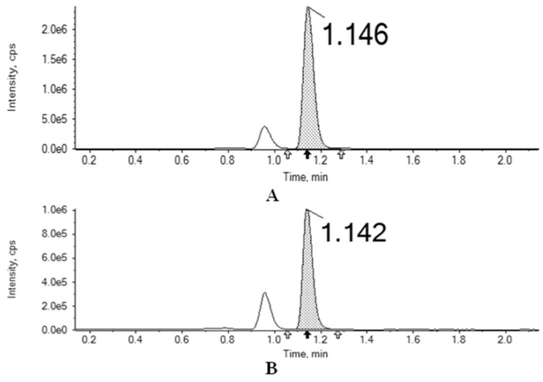

Figure S1. LC-MS of acrylamide extract from fried potato chips before and after L-asparaginase treatment. A: LC-MS of acrylamide extract diluted from fried potato chips without enzyme treatment; B: LC-MS of acrylamide extract diluted from fried potato chips treated with enzyme treatment.

Table S1. Biochemical characterization of L-asparaginases

Sources	Specific activity (IU/mg)	Optimum pH	Optimum T(°C)	Km (mM)	molecular weight (kDa)	Reference
A. niger	46.75	7.0	30	0.8141	90	[1]
P. fluorescens	26	7.5	37	50	35	[2]
V. cholerae	648.9	7.0	37	1.1	36.6	[3]
B. subtilis strain R5	250	9.0	35	2.4	60	[4]
S. brollosae	76.671	8.5	37	2.139	67	[5]
A. terreus	339.34	5.8	40	31.5	85	[6]
B. megaterium	215	8.5	37	0.2	47	[7]
B. licheniformis	36.08	7.0	37	49.995	37	[8]
B. amyloliquefaciens	4.75	8.5	65	1.5	38.2	[9]
B. altitudinis	800	7.0	37	90.9	35	[10]
C. amphilecti	778	7.0	60	2.05	37	[11]
T. kodakarensis	767	7.0	85	3.1	32.6	[12]
P. furiosus	11,203.5	8.0	50	1.623	33.66	[13]
B. sonorensis	4438.62	7.0	45	2.004	36	[14]

Table S2. Comparison of reduction in acrylamide formation in potato-based foods treated with L-asparaginase

Sources	Specific activity	Enzyme dosage	treatment and fried condition	Acrylamide reduction	reference
M. gordonis	486.7 IU/mg	40.0 IU/mL	37 ℃ 30 min<br>180 ℃ 5 min	65.09%	This study
Aquabacterium sp. A7-Y	458.9 IU/mg	14.6 IU/mL	45 ℃ 30 min<br>170 ℃ 5 min	93.4%	[15]
A. oryzae	NR	10.0 IU/mL	40 °C 15 min<br>180 ℃ 2.5 min	60%	[16]
B. megaterium	44.7 IU/mg	10.0 IU/mL	37 °C 1 h	92.4%	[17]
P. barengoltzii	35.2 IU/mg	80.0 IU/mL	45 ℃ 20 min<br>170 ℃ 5 min	86%	[18]
Cladosporium sp	83.3 IU/mg	50.0 IU/ mL	room temperature<br>40 min<br>180 ℃ 8 min	96%	[19]
Acinetobacter soli	400.0 IU/mg	30.0 IU/ mL	37℃ 30 min<br>170℃ 5 min	55.9%	[20]
B. subtilis	45.4 IU/mg	40.0 IU/ mL	60℃ 10 min<br>90℃ 20 min	Above 80%	[21]
T. zilligii AN1	5278.0 IU/mg	10.0 IU/ mL	80℃ 15 min<br>170℃ 5 min	85%	[22]

NR: not reported

References

1. Vala, A. K.; Sachaniya, B.; Dudhagara, D.; Panseriya, H. Z.; Gosai, H.; Rawal, R.; Dave, B. P. Characterization of L-asparaginase from marine-derived *Aspergillus niger* AKV-MKBU, its antiproliferative activity and bench scale production using industrial waste. *International Journal of Biological Macromolecules.* **2018,** 108, 41-46.

2. Sindhu, R.; Manonmani, H. K. Expression and characterization of recombinant L-asparaginase from *Pseudomonas fluorescens*. *Protein Expression and Purification.* **2018,** 143, 83-91.

3. Radha, R.; Arumugam, N.; Gummadi, S. N. Glutaminase free L-asparaginase from Vibrio cholerae: Heterologous expression, purification and biochemical characterization. *International Journal of Biological Macromolecules.* **2018,** 111, 129-138.

4. Chohan, S. M.; Rashid, N. Gene cloning and characterization of recombinant L-asparaginase from *Bacillus subtilis* strain R5. *Biologia.* **2018,** 73, 537-543.

5. El-Naggar, N. E.-A.; Deraz, S. F.; El-Ewasy, S. M.; Suddek, G. M. Purification, characterization and immunogenicity assessment of glutaminase free L-asparaginase from *Streptomyces brollosae* NEAE-115. *Bmc Pharmacology & Toxicology.* **2018,** 19, 51.

6. Hassan, S. W. M.; Farag, A. M.; Beltagy, E. A. Purification, characterization and anticancer activity of L-asparaginase produced by marine *Aspergillus terreus*. *Journal of Pure and Applied Microbiology.* **2018,** 12, 1845-1854.

7. Roy, M. P.; Das, V.; Patra, A. Isolation, purification and characterization of an extracellular L-asparaginase produced by a newly isolated *Bacillus megaterium* strain MG1 from the water bodies of Moraghat forest, Jalpaiguri, India. *Journal of General and Applied Microbiology.* **2019,** 65, 137-144.

8. Alrumman, S. A.; Mostafa, Y. S.; Al-Izran, K. A.; Alfaifi, M. Y.; Taha, T. H.; Elbehairi, S. E. Production and

anticancer activity of an L-asparaginase from *Bacillus licheniformis* isolated from the Red Sea, Saudi Arabia. *Scientific Reports.* **2019,** 9.

9. Yim, S.; Kim, M. Purification and characterization of thermostable L-asparaginase from *Bacillus amyloliquefaciens* MKSE in Korean soybean paste. *Lwt-Food Science and Technology.* **2019,** 109, 415-421.

10. Prakash, P.; Singh, H. R.; Jha, S. K. Production, purification and kinetic characterization of glutaminase free anti-leukemic L-asparaginase with low endotoxin level from novel soil isolate. *Preparative Biochemistry & Biotechnology.* **2020,** 50, 260-271.

11. Farahat, M. G.; Amr, D.; Galal, A. Molecular cloning, structural modeling and characterization of a novel glutaminase-free L-asparaginase from *Cobetia amphilecti* AMI6. *International Journal of Biological Macromolecules.* **2020,** 143, 685-695.

12. Chohan, S. M.; Sajed, M.; Naeem, S. U.; Rashid, N. Heterologous gene expression and characterization of TK2246, a highly active and thermostable plant type L-asparaginase from *Thermococcus kodakarensis*. *International Journal of Biological Macromolecules.* **2020,** 147, 131-137.

13. Saeed, H.; Hemida, A.; El-Nikhely, N.; Abdel-Fattah, M.; Shalaby, M.; Hussein, A.; Eldoksh, A.; Ataya, F.; Aly, N.; Labrou, N.; Nematalla, H. Highly efficient *Pyrococcus furiosus* recombinant L-asparaginase with no glutaminase activity: Expression, purification, functional characterization, and cytotoxicity on THP-1, A549 and Caco-2 cell lines. *International Journal of Biological Macromolecules.* **2020,** 156, 812-828.

14. Aly, N.; El-Ahwany, A.; Ataya, F. S.; Saeed, H. *Bacillus sonorensis* L. Asparaginase: Cloning, expression in *E. coli* and characterization. *Protein Journal.* **2020,** 39, 717-729.

15. Sun, Z.; Qin, R.; Li, D.; Ji, K.; Wang, T.; Cui, Z.; Huang, Y. A novel bacterial type II L-asparaginase and evaluation of its enzymatic acrylamide reduction in French fries. *International Journal of Biological Macromolecules.* **2016,** 92, 232-239.

16. Hendriksen, H. V.; Kornbrust, B. A.; Ostergaard, P. R.; Stringer, M. A. Evaluating the potential for enzymatic acrylamide mitigation in a range of food products using an asparaginase from *Aspergillus oryzae*. *Journal of Agricultural and Food Chemistry.* **2009,** 57, 4168-4176.

17. Zhang, S.; Xie, Y.; Zhang, C.; Bie, X.; Zhao, H.; Lu, F.; Lu, Z. Biochemical characterization of a novel L-asparaginase from *Bacillus megaterium* H-1 and its application in French fries. *Food Research International.* **2015,** 77, 527-533.

18. Shi, R.; Liu, Y.; Mu, Q.; Jiang, Z.; Yang, S. Biochemical characterization of a novel L-asparaginase from *Paenibacillus barengoltzii* being suitable for acrylamide reduction in potato chips and mooncakes. *International Journal of Biological Macromolecules.* **2017,** 96, 93-99.

19. Kumar, N. S. M.; Manonmani, H. K. Purification, characterization and kinetic properties of extracellular L-asparaginase produced by *Cladosporium* sp. *World Journal of Microbiology & Biotechnology.* **2013,** 29, 577-587.

20. Jiao, L.; Chi, H.; Lu, Z.; Zhang, C.; Chia, S. R.; Show, P. L.; Tao, Y.; Lu, F. Characterization of a novel type I L-asparaginase from *Acinetobacter soli* and its ability to inhibit acrylamide formation in potato chips. *Journal of Bioscience and Bioengineering.* **2020,** 129, 672-678.

21. Onishi, Y.; Prihanto, A. A.; Yano, S.; Takagi, K.; Umekawa, M.; Wakayama, M. Effective treatment for suppression of acrylamide formation in fried potato chips using L-asparaginase from *Bacillus subtilis*. *3 Biotech.* **2015,** 5, 783-789.

22. Zuo, S.; Zhang, T.; Jiang, B.; Mu, W. Reduction of acrylamide level through blanching with treatment by an extremely thermostable L-asparaginase during French fries processing. *Extremophiles.* **2015,** 19, 841-851.
